# The Prognostic Value of Preoperative Systemic Inflammatory Response Index (SIRI) in Patients With High-Grade Glioma and the Establishment of a Nomogram

**DOI:** 10.3389/fonc.2021.671811

**Published:** 2021-05-14

**Authors:** Qian He, Longhao Li, Qinglan Ren

**Affiliations:** Department of Oncology, The First Affiliated Hospital of Chongqing Medical University, Chongqing, China

**Keywords:** systemic inflammatory response index, high-grade glioma, overall survival, nomogram, prognosis prediction

## Abstract

**Background:**

The predictive value of systemic inflammatory response index (SIRI) was confirmed in some malignant tumors. However, few studies investigated the prognostic value of SIRI in high-grade gliomas. This study aimed to evaluate the prognostic relationship of preoperative SIRI in high-grade gliomas and established a nomogram accordingly.

**Methods:**

Data of operable high-grade glioma patients were analyzed. Kaplan-Meier, log-rank test, cox regression and propensity score matching (PSM) analysis were used to analyze survival. ROC curve and area under the curve (AUC) were used to compare the ability of preoperative SIRI, neutrophil-lymphocyte ratio (NLR), platelet-lymphocyte ratio (PLR) and monocyte-lymphocyte ratio (MLR) to predict prognosis. A nomogram based on the results was established. The consistency index (C-index) was calculated and a calibration curve was drawn.The prediction effect of the nomogram and WHO grade was compared by AUC.

**Results:**

A total of 105 patients were included. Kaplan-Meier survival analysis showed that the overall survival (OS) of grade III gliomas patients with lower preoperative SIRI (SIRI<1.26) was significantly prolonged (p=0.037), and grade IV gliomas patients with lower preoperative SIRI had a tendency to obtain longer OS (p = 0.107). Cox regression showed preoperative SIRI was an independent prognostic factor for grade IV and grade III glioma, however, in IDH mutant-type IV gliomas, patients with lower SIRI only showed a tendency to obtain better OS. Similar results were obtained in PSM. The prognostic value of SIRI were better than PLR and MLR by ROC analysis. And in grade IV gliomas, the predictive value of SIRI was better than NLR. The nomogram established based on preoperative SIRI, age, extent of resection, number of gliomas, MGMT methylation status and histological types (only in grade III gliomas) could predict the prognosis more accurately.

**Conclusion:**

SIRI was valuable for prognosis prediction in high-grade glioma. The nomogram covering SIRI could more accurately predict the survival rate in operable high-grade glioma patients.

## Introduction

High-grade glioma is a common type of primary brain cancer, accounting for more than half of primary central nervous system malignancies (1, 2). The current standard treatment for high-grade glioma is surgery, radiotherapy, temozolomide adjuvant chemotherapy, tumor treating fields, etc. (3, 4). The overall prognosis of high-grade glioma is poor, but we can see that the survival time of patients with high-grade glioma is quite different. We need to find favorable markers to predict the prognosis of patients. Patient’s age, tumor grade and molecular characteristics are commonly used in clinical to predict the prognosis of patients with high-grade glioma (2). Even so, we cannot accurately predict the prognosis of patients, and more indicators are needed.

Tumor-related inflammation is closely related to anti-tumor effects (5). More and more evidence showed that inflammatory response affected the growth, progression, metastasis and other stages of cancer, as well as immune surveillance and treatment response (6). Studies showed that systemic inflammatory markers such as neutrophil-lymphocyte ratio (NLR), monocyte-lymphocyte ratio (MLR) and platelet-lymphocyte ratio (PLR) were important for predicting the prognosis of many kinds of malignant tumors (7–12). SIRI is an inflammation marker with simple detection, low cost and strong practicability. It is calculated as: neutrophil count × monocyte count/lymphocyte count. It is based on neutrophils, monocytes and lymphocytes, and can more comprehensively evaluate the relationship between anti-tumor immune effects and inflammation. Its predictive value has been confirmed in a variety of malignant tumors (13–15). However, there were few studies on the prognostic value of SIRI in gliomas. This may be related to the recognition that the central nervous system (CNS) has immune privilege due to the existence of the blood-brain barrier (BBB) and the lack of lymphatic vessels in the CNS parenchyma (16). However, this view is constantly being updated. In some brain tumors, the BBB may be damaged, allowing a number of immune cells in the peripheral blood to infiltrate (17). Some studies showed that NLR in peripheral blood may be related to the prognosis of patients with glioma (10, 18). Based on the findings above, this study aimed to explore the prognostic value of preoperative SIRI, NLR, MLR, and PLR in patients with high-grade glioma surgery.

## Material and Methods

### Patients

We retrospectively analyzed the clinical data of patients with high-grade glioma who underwent surgery in the First Affiliated Hospital of Chongqing Medical University from December 2013 to December 2019. The inclusion criteria were as follows (1): patients who were pathologically diagnosed as high-grade glioma, based on 2016 WHO classification, global standard, after surgical resection; (2) patients who completed the “Stupp” regimen of chemoirridiation; (3) patients with complete follow-up data; (4) patients with blood routine examination before the use of steroid and within 1 week before operation. The exclusion criteria were as follows: (1) patients with incomplete data; (2) patients who received neoadjuvant chemotherapy or radiotherapy before surgery; (3) patients who did not receive adjuvant radiotherapy and chemotherapy after surgery; (4) patients with a history of infection or inflammatory diseases in the past month. Finally, survival analysis was performed on the collected follow-up data of patients. Due to the use of unidentified patient data, the study was exempted by the Ethics Committee of the First Affiliated Hospital of Chongqing Medical University.

### Data Collection and Hematological Examination

Demographic and clinicopathological data include patient age, sex, WHO grade, histological type, tumor location, etc. Based on the preoperative and postoperative MRI, as well as the surgeon’s observation during the operation, gross total resection (GTR) was defined as no tumor remaining, near total resection (NTR) was defined as> 90% resection, and subtotal resection (STR) was defined as 80-90% resection, and partial resection (PR) was defined as a resection rate of <80%. The blood routines were collected 1 week before operation, including neutrophil count, monocyte count, lymphocyte count, and platelet count. The definitions of SIRI, NLR, MLR and PLR were as follows: SIRI=neutrophil count*monocyte count/lymphocyte count; NLR=neutrophil count/lymphocyte count; MLR, monocyte count/lymphocyte count; PLR, platelet count/lymphocyte count. Use X-tile software to find the optimal cutoff values of SIRI, NLR, PLR and MLR in the queue. The best cut-off values were as follows: SIRI (1.26), NLR (3.31), MLR (0.20), PLR (194).

### Follow Up

The primary end point was OS. A total of 198 newly diagnosed high-grade glioma patients underwent surgery in our hospital. Among them, 39 patients were recently lost to follow-up because of not updating the latest phone number, and 54 patients were excluded because they did not complete the “Stupp” regimen of chemoirridiation. Finally, a total of 105 cases were included and followed up successfully. OS was defined from the day of surgery to the death of the patient or the final follow-up time. The follow-up ended on September 15, 2020.

### Statistical Analysis

Continuous variables were compared by independent sample T test or Mann-Whitney U test. Categorical variables were compared by χ2 test or Fisher’s exact probability test. The Kaplan-Meier method was used to analyze the correlation between variables and overall survival, and the log-rank test was used to compare survival curves. The Cox regression model was used for univariate and multivariate survival analysis, and the Cox proportional hazard model was used to calculate the hazard ratio (HR) and 95% confidence interval (CI). Since we were more concerned about the relationship between SIRI and the prognosis of high-grade gliomas, among all inflammatory factors, we only included SIRI in multivariate survival analysis. In addition, due to the imbalance of baseline characteristics, the nearest neighbor matching algorithm was used for PSM analysis, allowing the maximum tolerance of propensity score to be less than 20% (in grade IV gliomas) and 30% (in grade IV gliomas, due to small sample size) of the propensity score SD. Through the ROC curve, combined with specificity and sensitivity, the area under the curve (AUC) is measured and compared to evaluate the prognostic ability of SIRI, NLR, PLR, and MLR. We established a nomogram based on the statistically significant results in the multivariate analysis. And we calculated the C-index. Calibration curve were performed to verify the predictive nomogram through bootstrap sampling 1,000 times. The ROC curve was used to compare the value of nomogram and WHO in predicting OS. SPSS version 25, X-tile version 3.6.1 and R version 4.0.2 statistical software were used for data analysis. All P values are two-sided, with statistical significance set to 0.05.

## Results

### Patient Characteristics

A total of 105 patients with high-grade glioma were included. The median age was 50 years (18-79 years). Among them, 43 cases were grade III gliomas and 62 cases were grade IV gliomas. All patients received the “Stupp” regimen of chemoirridiation. The clinical and pathological characteristics of grade 4 glioma patients and grade III glioma patients were shown in **Tables 1** and **2** (complete dataset and 1:1 matched dataset). The correlation between SIRI and clinicopathological characteristics was also shown in **Tables 1** and **2**.

**Table 1:** Baseline patient characteristics stratified by inflammatory marker levels in grade IV glioma.

Variables	Complete dataset					1:1 matched dataset				
	Total (n=62)	%	SIRI ≤1.26 (n=29, 46.8%)	SIRI >1.26 (n=33, 53.2%)	p	Total (n=48)	%	SIRI ≤1.26 (n=24, 50.0%)	SIRI >1.26 (n=24, 50.0%)	p
	N/ M ± SD		N/ M ± SD	N/ M ± SD		N/ M ± SD		N/ M ± SD	N/ M ± SD	
**Age**	50.44 ± 14.642		50.41 ± 16.961	50.45 ± 12.528	0.991	52.04 ± 14.244		51.39 ± 16.303	52.70 ± 12.178	0.760
**Sex**										
female	32	51.6%	14	18	0.622	24	52.2%	12	12	1.000
male	30	48.4%	15	15		22	47.8%	11	11	
**Main location**										
frontal	37	59.7%	16	21	0.498	27	58.7%	13	14	0.765
parietal	20	32.3%	10	10	0.725	16	34.8%	9	7	0.536
occipital	11	17.7%	6	5	0.569	8	17.4%	4	4	1.000
temporal	13	21.0%	7	6	0.565	8	17.4%	5	3	0.699
insular	2	3.2%	0	2	0.494	2	4.3%	0	2	0.489
other	9	14.5%	4	5	1.000	7	15.2%	4	3	1.000
**No. of glioma**										
single	55	88.7%	25	30	0.696	41	89.1%	21	20	1.000
**multiple**	7	11.3%	4	3		5	10.9%	2	3	
**Extent of resection**										
PR	1	1.6%	0	1	0.112	1	2.2%	0	1	0.177
STR	7	11.3%	4	3		6	13.0%	4	2	
NTR	8	12.9%	1	7		6	13.0%	1	5	
GTR	46	74.2%	24	22		33	71.7%	18	15	
**IDH mutation**										
no	42	67.7%	23	19	0.068	35	76.1%	17	18	0.730
yes	20	32.3%	6	14		11	23.9%	6	5	
**MGMT methylation**										
no	41	66.1%	22	19	0.129	32	69.6%	16	16	1.000
yes	21	33.9%	7	14		14	30.4%	7	7	
**1p19q deletion**										
no	62	100%	29	33	NA	46	100%	23	23	NA
yes	0	0%	0	0		0	0.0%	0	0	
**ATRX mutation**										
no	56	90.3%	24	32	0.089	42	91.3%	20	22	0.608
yes	6	9.7%	5	1		4	8.7%	3	1	
**TP53**										
negative	20	32.3%	12	8	0.150	15	32.6%	10	5	0.116
positive	42	67.7%	17	25		31	67.4%	13	18	
**Ki-67**	28.11 ± 14.724		29.11 ± 13.747	27.27 ± 15.667	0.632	28.04 ± 14.317		27.61 ± 14.051	28.48 ± 14.881	0.839
**Epilepsy before surgery**										
no	51	82.3%	24	27	0.923	37	80.4%	20	17	0.459
yes	11	17.7%	5	6		9	19.6%	3	6	

GTR, gross total resection; M, mean; N, number; NA, not applicable; No., number; NTR, near total resection; PR, partial resection; SD, standard deviation; SIRI, systemic inflammatory response index; STR, subtotal resection.

**Table 2:** Baseline patient characteristics stratified by inflammatory marker levels in grade III glioma.

Variables	Complete dataset					1:1 matched dataset				
	Total (n=43)	%	SIRI ≤1.26 (n=25, 58.1%	SIRI >1.26 (n=18, 41.9%)	p	Total (n=30)	%	SIRI ≤1.26 (n=15, 50.0%)	SIRI >1.26 (n=15, 50.0%)	p
	N/ M ± SD		N/ M ± SD	N/ M ± SD		N/ M ± SD		N/ M ± SD	N/ M ± SD	
**Age**	48.56 ± 11.189		48.40 ± 10.388	48.78 ± 12.526	0.915	47.36 ± 10.393		46.43 ± 7.623	48.29 ± 12.821	0.645
**Sex**										
female	16	37.2%	10	6	0.655	12	42.9%	6	6	1.000
male	27	62.8%	15	12		16	57.1%	8	8	
**Histology**										
AA	23	53.5%	15	8	0.598	16	57.1%	8	8	1.000
AO	18	41.9%	9	9		12	42.9%	6	6	
NOS	2	4.7%	1	1		0	0.0%	0	0	
**Main location**										
frontal	30	69.8%	19	11	0.294	19	67.9%	10	9	1.000
parietal	5	11.6%	3	2	1.000	3	10.7%	2	1	1.000
occipital	3	7.0%	2	1	1.000	2	7.1%	1	1	1.000
temporal	13	30.2%	6	7	0.294	8	28.6%	3	5	0.678
insular	3	7.0%	2	1	1.000	2	7.1%	1	1	1.000
other	5	11.6%	2	3	0.634	4	14.3%	1	3	0.596
**No. of glioma**										
single	41	95.3%	24	17	1.000	27	96.4%	14	13	1.000
multiple	2	4.7%	1	1		1	3.6%	0	1	
**Extent of resection**										
PR	3	7.0%	1	2	0.567	2	7.1%	0	2	0.162
STR	4	9.3%	3	1		3	10.7%	2	1	
NTR	5	11.6%	4	1		3	10.7%	3	0	
GTR	31	72.1%	17	14		20	71.4%	9	11	
**IDH mutation**										
no	5	11.6%	4	1	0.380	2	7.1%	1	1	1.000
yes	38	88.4%	21	17		26	92.9%	13	13	
**MGMT methylation**										
no	20	46.5%	12	8	0.818	14	50.0%	7	7	1.000
yes	23	53.5%	13	10		14	50.0%	7	7	
**1p19q deletion**										
no	25	58.1%	16	9	0.359	16	57.1%	8	8	1.000
yes	18	41.9%	9	9		12	42.9%	6	6	
**ATRX mutation**										
no	25	58.1%	13	12	0.336	15	53.6%	7	8	0.705
yes	18	41.9%	12	6		13	46.4%	7	6	
**TP53**										
negative	7	16.3%	4	3	1.000	3	10.7%	1	2	1.000
positive	36	83.7%	21	15		25	89.3%	13	12	
**Ki-67**	18.02 ± 16.246		14.68 ± 13.388	22.67 ± 18.967	0.113	16.11 ± 13.796		13.07 ± 11.256	19.14 ± 15.772	0.252
**Epilepsy before surgery**										
no	27	62.8%	15	12	0.655	15	53.6%	6	9	0.256
yes	16	37.2%	10	6		13	46.4%	8	5	

AA, anaplastic astrocytomas; AO, anaplastic oligodendrogliomas; GBM, glioblastoma; GTR, gross total resection; M, mean; N, number; NA, not applicable; No., number; NOS, not otherwised speccified; NTR, near total resection; PR, partial resection; SD, standard deviation; SIRI, systemic inflammatory response index; STR, subtotal resection.

### Survival Analysis

The median OS for all patients was 20 months (95% CI: 21.7-29.0 months). The median survival for grade III glioma is 22 months (95% CI: 25.9-37.6 months), and 15 months (95% CI: 16.5-25.3 months) for grade IV glioma. The median survival for preoperative low SIRI (≤1.26) group was 22 months (95% CI: 23.4-34.0 months), and 17 months (95% CI: 16.9-26.7 months) for the preoperative high SIRI (>1.26) group. In the preoperative low NLR (≤3.31) group, the median survival was 21 months (95% CI: 22.8-32.1 months), and in the preoperative high NLR (>3.31) group, it was 15 months (95% CI: 16.1-27.8 months). The median survival of patients in the preoperative low MLR (≤0.20) group and high MLR (>0.20) group were 24 months (95% CI: 22.3-38.0 months) and 18 months (95% CI: 19.6-27.8 months), respectively. And the median survival for the preoperative low PLR (≤194) group and high PLR (>194) were 21 months (95% CI: 22.5-30.3 months) and 14 months (95% CI: 9.6-28.7 months), respectively.

**Figure 1:**
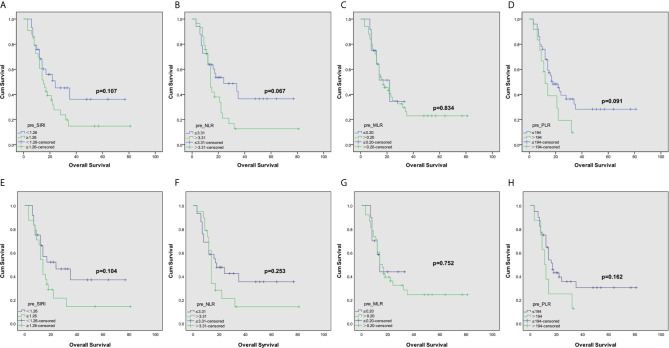
Kaplan–Meier curves for patients stratified based on **(A)** SIRI, **(B)** NLR, **(C)** MLR and **(D)** PLR in operable grade IV glioma patients in complete dataset. And Kaplan–Meier curves for patients stratified based on **(E)** SIRI, **(F)** NLR, **(G)** MLR and **(H)** PLR in operable grade IV glioma patients after propensity matching.

**Figure 2:**
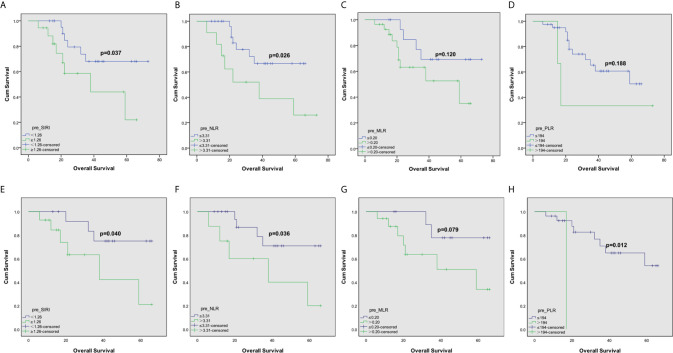
Kaplan–Meier curves for patients stratified based on **(A)** SIRI, **(B)** NLR, **(C)** MLR and **(D)** PLR in operable grade III glioma patients in complete dataset. And Kaplan–Meier curves for patients stratified based on **(E)** SIRI, **(F)** NLR, **(G)** MLR and **(H)** PLR in operable grade III glioma patients after propensity matching.

The Kaplan-Meier survival curve of grade IV gliomas in the complete dataset was shown in **Figures 1A–D**. Grade IV gliomas patients with lower SIRI tended to have better OS (p=0.107). Among matched patients, similar survival advantage trend was also found in patients with lower preoperatively SIRI (p=0.083, **Figures 1E–H**). In grade III gliomas, the OS significantly prolonged in lower SIRI group in the complete dataset (P =0.037, **Figures 2A–D**). This was also shown in the matched dataset (p=0.040, **Figures 2E–H**).

In the unmatched complete dataset of grade IV glioma, univariate survival analysis showed that patients with lower preoperative SIRI tended to have better prognosis (HR= 1.661, p=0.119, **Table 3**). And the multivariate analysis suggested that preoperative SIRI level was an independent prognostic factor in grade IV glioma (HR= 3.963, p<0.001, **Table 3**). In the 1:1 matched dataset, multivariate analysis also showed that preoperative SIRI level was a statistically significant impact on OS (HR= 3.204, p=0.006, **Table 3**). We further divided grade IV glioma patients into two subgroups: IDH wild-type and mutant-type to distinguish primary or secondary grade IV glioma (19). The predictive value of preoperative SIRI in the two subgroups may be different. Our results showed that in the IDH wild-type IV glioma subgroup (n=42), preoperative SIRI was an independent prognostic factor of OS (HR=2.814, P=0.020, **Table 4**), while in IDH mutant-type subgroup (n=20), only similar trends were found (HR=13.234, p=0.071, **Table 4**). In grade III glioma, the preoperative SIRI level was an independent prognostic factor in the complete dataset and the matched dataset (HR=36.973, p=0.003 and HR=12.043, p=0.024, respectively, **Table 5**). Among grade III gliomas, IDH wild-type patients may be classified as grade IV gliomas after further testing. Therefore, we conducted further analysis on patients with IDH mutant-type patients in grade III glioma. The results showed that whether in univariate analysis or multivariate analysis, the OS advantage of patients with lower preoperative SIRI in IDH mutant-type subgroup in grade III glioma was statistically significant (HR=3.711, p=0.024 and HR=24.479, p=0.001, respectively, **Table 6**). Due to the small number of IDH wild-type patients in grade III gliomas (n=5), we did not further analyze them. Looking forward to the subsequent inclusion of more patients for further research.

**Table 3:** Univariate and multivariate cox regression analyses for overall survival in grade IV glioma.

Variable	Complete dataset								1:1 matched dataset							
	Univariate analysis				Multivariate analysis				Univariate analysis				Multivariate analysis			
	HR	(95% CI)		p value	HR	(95% CI)		p value	HR	(95% CI)		p value	HR	(95% CI)		p value
**Age**	1.018	(0.996-	1.040)	0.103	1.006	0.983	1.028	0.624	1.016	0.992	1.041	0.188	1.007	0.982	1.032	0.600
**Sex**																
male vs. female	1.152	(0.624-	2.128)	0.65					1.183	0.584	2.396	0.640				
**No. of glioma**																
multiple vs. single	3.806	(1.543-	9.386)	0.004*	3.755	1.303	10.818	0.014*	7.459	2.559	21.741	<0.001*	6.826	1.942	23.994	0.003*
**Extent of resection**																
GTR+NTR vs. STR+PR	0.21	0.087	0.508	0.001*	0.153	0.057	0.412	<0.001*	0.237	0.09	0.619	0.003*	0.209	0.07	0.622	**0.005**
**IDH mutation**																
yes vs. no	0.518	(0.260-	1.033)	0.062	0.631	0.271	1.466	0.284	0.372	0.14	0.984	0.046*	0.38	0.127	1.132	0.082
**MGMT methylation**																
positive vs. negative	0.295	(0.141-	0.615)	0.001*	0.247	0.096	0.634	0.004*	0.261	0.106	0.643	0.003*	0.318	0.115	0.878	0.027*
**ATRX mutation**																
yes vs. no	0.681	(0.209-	2.222)	0.525					0.817	0.246	2.713	0.742				
**TP53**																
yes vs. no	0.725	(0.378-	1.387)	0.331					0.883	0.415	1.879	0.747				
**Ki-67**	1.003	(0.982-	1.023)	0.802					1.003	0.978	1.028	0.832				
**Epilepsy before surgery**																
yes vs. no	1.102	(0.484-	2.509)	0.818					1.116	0.452	2.755	0.812				
**preoperative SIRI**																
>1.26 vs. ≤1.26	1.661	(0.878-	3.141)	0.119	3.963	1.833	8.565	<0.001*	1.843	0.895	3.797	0.097	3.204	1.391	7.379	0.006*
**preoperative NLR**																
>3.31 vs. ≤3.31	1.766	(0.941-	3.316)	0.077					1.501	0.736	3.061	0.264				
**preoperative MLR**																
>0.20 vs. ≤0.20	1.095	(0.457-	2.623)	0.838					0.999	0.407	2.449	0.998				
**preoperative PLR**																
>194 vs. ≤194	1.819	(0.885-	3.742)	0.104					1.631	0.725	3.667	0.237				

CI, confidence interval; GTR, gross total resection; HR, hazard ratio; MLR, monocyte-lymphocyte ratio; NLR, neutrophil-lymphocyte ratio; No., number; NTR, near total resection; PLR, platelet-lymphocyte ratio; PR, partial resection; SIRI, systemic inflammatory response index; STR, subtotal resection. The bold values and the sign “*” meant: statistically significant (P < 0.05).

**Table 4:** Univariate and multivariate cox regression analyses for overall survival in IDH wild-type IV glioma subgroup and IDH mutant-type IV glioma subgroup.

Variable	IDH wild-type IV glioma subgroup								IDH mutant-type IV glioma subgroup							
	Univariate analysis				Multivariate analysis				Univariate analysis				Multivariate analysis			
	HR	(95% CI)		p value	HR	(95% CI)		p value	HR	(95% CI)		p value	HR	(95% CI)		p value
**Age**	1.007	(0.984-	1.030)	0.565	1.001	0.974	1.028	0.962	1.042	(0.994-	1.093)	0.086	1.029	0.975	1.085	0.297
**Sex**																
male vs. female	1.132	(0.540-	2.375)	0.742					0.787	(0.235-	2.634)	0.698				
**No. of glioma**																
multiple vs. single	2.669	(0.996-	7.153)	0.051	3.227	1.011	10.301	0.048*	24.486	(1.498-	400.306)	**0.025**	3.064	0.109	85.748	0.510
**Extent of resection**																
GTR+NTR vs. STR+PR	0.265	0.097	0.719	0.009*	0.176	0.058	0.533	0.002*	0.059	0.005	0.702	0.025*	0.047	0.001	1.585	0.088
**MGMT methylation**																
positive vs. negative	0.402	(0.136-	1.183)	0.098	0.36	0.11	1.174	0.090	0.133	(0.023-	0.750)	0.022*	0.115	0.017	0.784	0.027*
**ATRX mutation**																
yes vs. no	0.471	(0.142-	1.563)	0.219					NA							
**TP53**																
yes vs. no	1.117	(0.531-	2.354)	0.770					0.101	(0.014-	0.726)	0.023*				
**Ki-67**	0.996	(0.974-	1.020)	0.762					1.018	(0.977-	1.062)	0.391				
**Epilepsy before surgery**																
yes vs. no	2.644	(1.030-	6.785)	0.043*	1.981	0.719	5.453	0.186	0.205	(0.025-	1.665)	0.138				
**preoperative SIRI**																
>1.26 vs. ≤1.26	2.158	(1.004-	4.639)	0.049*	2.814	1.174	6.747	0.020*	2.128	(0.464-	9.752)	0.331	13.234	0.803	218.042	0.071
**preoperative NLR**																
>3.31 vs. ≤3.31	1.953	(0.918-	4.155)	0.082					2.276	(0.640-	8.091)	0.204				
**preoperative MLR**																
>0.20 vs. ≤0.20	1.078	(0.410-	2.834)	0.879					1.414	(0.177-	11.288)	0.744				
**preoperative PLR**																
>194 vs. ≤194	2.097	(0.842-	5.219)	0.112					2.247	(0.632-	7.995)	0.211				

CI, confidence interval; GTR, gross total resection; HR, hazard ratio; MLR, monocyte-lymphocyte ratio; NA, not applicable; NLR, neutrophil-lymphocyte ratio; No., number; NTR, near total resection; PLR, platelet-lymphocyte ratio; PR, partial resection; SIRI, systemic inflammatory response index; STR, subtotal resection. The bold values and the sign “*” meant: statistically significant (P < 0.05).

**Table 5:** Univariate and multivariate cox regression analyses for overall survival in grade III glioma.

Variable	Complete dataset								1:1 matched dataset							
	Univariate analysis				Multivariate analysis				Univariate analysis				Multivariate analysis			
	HR	(95% CI)		p value	HR	(95% CI)		p value	HR	(95% CI)		p value	HR	(95% CI)		p value
**Age**	1.077	(1.020-	1.137)	0.007*	1.177	1.072	1.292	0.001*	1.037	0.966	1.113	0.315	1.162	1.036	1.303	0.010*
**Sex**																
male vs. female	1.042	(0.348-	3.119)	0.942					0.832	0.222	3.119	0.785				
**Histology**																
AO vs. AA	0.659	0.203	2.146	0.489	0.065	0.008	0.545	0.012*	0.202	0.025	1.62	0.132	0.082	0.007	0.893	0.040*
NOS vs. AA	1.209	0.151	9.698	0.859	0.013	0	3.256	0.123	NA							
**No. of glioma**																
multiple vs. single	6.283	(1.328-	29.73)	0.020*	2.432	0.181	32.743	0.503	25.456	1.59	407.465	0.022*	15.044	0.268	845.737	0.187
**Extent of resection**																
GTR+NTR vs. STR+PR	0.284	0.093	0.871	0.028*	0.085	0.011	0.649	0.018*	0.214	0.053	0.862	0.030*	0.079	0.009	0.678	0.021*
**IDH mutation**																
yes vs. no	2.248	0.293	17.221	0.436	0.452	0.036	5.691	0.539	24.962	0.004	158584.761	0.471				
**MGMT methylation**																
positive vs. negative	0.516	(0.176-	1.517)	0.229	0.022	0.002	0.308	0.005*	0.665	0.176	2.513	0.548	0.08	0.007	0.929	0.044*
**1p19q deletion**																
yes vs. no	0.648	(0.203-	2.074)	0.465					0.202	0.025	1.62	0.132				
**ATRX mutation**																
yes vs. no	1.484	(0.512-	4.298)	0.467					7.132	0.888	57.285	0.065				
**TP53**																
yes vs. no	0.51	(0.111-	2.333)	0.385					23.16	0	3.95091E+11	0.794				
**Ki-67**	1.038	(1.002-	1.075)	0.036*	1.049	0.964	1.141	0.264	1.031	0.98	1.086	0.238				
**Epilepsy before surgery**																
yes vs. no	0.317	(0.088-	1.147)	0.08	0.276	0.049	1.564	0.146	0.374	0.092	1.512	0.168				
**preoperative SIRI**																
>1.26 vs. ≤1.26	2.935	(1.009-	8.540)	0.048*	36.973	3.411	400.783	0.003*	3.981	0.968	16.371	0.055	12.043	1.381	104.989	0.024*
**preoperative NLR**																
>3.31 vs. ≤3.31	3.11	(1.082-	8.938)	0.035*					3.735	0.997	13.994	0.051				
**preoperative MLR**																
>0.20 vs. ≤0.20	2.456	(0.759-	7.944)	0.134					3.773	0.772	18.439	0.101				
**preoperative PLR**																
>194 vs. ≤194	2.654	(0.582-	12.100)	0.207					11.505	1.037	127.618	0.047*				

AA, anaplastic astrocytomas; AO, anaplastic oligodendrogliomas; CI, confidence interval; GTR, gross total resection; HR, hazard ratio; MLR, monocyte-lymphocyte ratio; NA, not applicable; NLR, neutrophil-lymphocyte ratio; No., number; NOS, not otherwised speccified; NTR, near total resection; PLR, platelet-lymphocyte ratio; PR, partial resection; SIRI, systemic inflammatory response index; STR, subtotal resection. The bold values and the sign “*” meant: statistically significant (P < 0.05).

**Table 6:** Univariate and multivariate cox regression analyses for overall survival in IDH mutant-type grade III glioma subgroup.

Variable	Univariate analysis				Multivariate analysis			
	HR	(95% CI)		p value	HR	(95% CI)		p value
**Age**	1.067	(1.010-1.127)		0.020*	1.147	1.058	1.244	0.001*
**Sex**								
male vs. female	1.355	(0.442-4.155)		0.596				
**Histology**								
AO vs. AA	0.552	0.166 1.839		0.333	0.153	0.031	0.747	0.020*
NOS vs. AA	0.987	0.1218.042		0.990	0.258	0.014	4.851	0.366
**No. of glioma**								
multiple vs. single	5.735	(1.185-27.766)		0.030*	7.732	0.686	87.216	0.098
**Extent of resection**								
GTR+NTR vs. STR+PR	0.283	0.089 0.898		0.032*	0.152	0.025	0.914	0.040*
**MGMT methylation**								
positive vs. negative	0.503	(0.165-1.530)		0.226	0.042	0.005	0.364	0.004*
**1p19q deletion**								
yes vs. no	0.652	(0.200- 2.123)		0.478				
**ATRX mutation**								
yes vs. no	1.651	(0.538- 5.059)		0.381				
**TP53**								
yes vs. no	0.563	(0.123-2.572)		0.458				
**Ki-67**	1.037	(1.000-1.075)		0.050				
**Epilepsy before surgery**								
yes vs. no	0.326	(0.089-1.198)		0.091				
**preoperative SIRI**								
>1.26 vs. ≤1.26	3.711	(1.191-11.565)		0.024*	24.479	3.647	164.311	0.001*
**preoperative NLR**								
>3.31 vs. ≤3.31	3.534	(1.183-10.561)		0.024*				
**preoperative MLR**								
>0.20 vs. ≤0.20	2.847	(0.765-10.594)		0.119				
**preoperative PLR**								
>194 vs. ≤194	2.217	(0.482-10.196)		0.306				

AA, anaplastic astrocytomas; AO, anaplastic oligodendrogliomas; CI, confidence interval; GTR, gross total resection; HR, hazard ratio; MLR, monocyte-lymphocyte ratio; NLR, neutrophil-lymphocyte ratio; No., number; NOS, not otherwised speccified; NTR, near total resection; PLR, platelet-lymphocyte ratio; PR, partial resection; SIRI, systemic inflammatory response index; STR, subtotal resection. The bold values and the sign “*” meant: statistically significant (P < 0.05).

**Figure 3:**
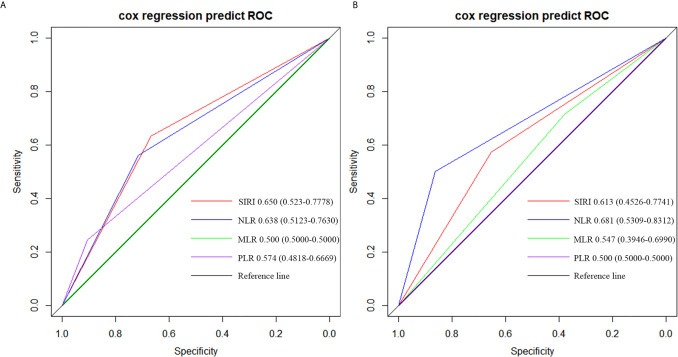
Predictive ability of the SIRI, NLR, MLR, and PLR in operable **(A)** grade IV and **(B)** grade III glioma patients by ROC curves.

By comparing the AUC value of the ROC curve to judge the predictive power of SIRI, NLR, MLR and PLR for patient survival, we found that in grade IV glioma, the AUC of SIRI (AUC = 0.650) was greater than NLR (AUC = 0.638), PLR (AUC = 0.574) and MLR (AUC = 0.500), which indicated that the prognostic value of SIRI were better than NLR, PLR and MLR (**Figure 3A**). In grade III glioma, the AUC of SIRI (AUC = 0.613) was greater than MLR (AUC = 0.547) and PLR (AUC = 0.500), but lower than NLR (AUC = 0.681). This indicated that the prognostic value of NLR may be better than SIRI in grade III glioma (**Figure 3B**).

### The Establishment of a Nomogram

**Figure 4:**
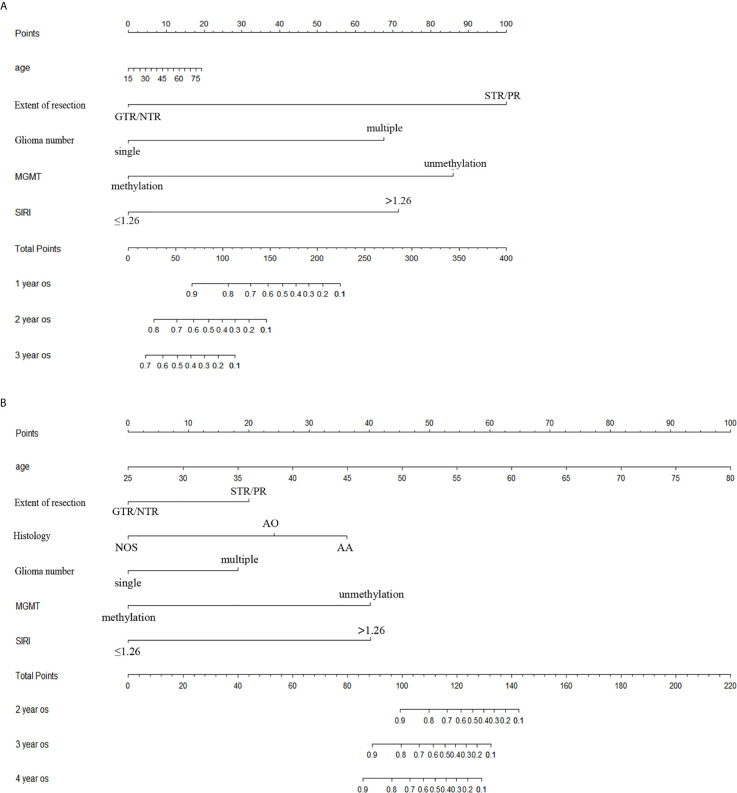
The nomogram **(A)** covering age, extent of resection, number of glioma, MGMT methylation status and SIRI at 1-year, 2-year and 3-year in operable grade IV glioma patients and **(B)** covering age, extent of resection, histological type, number of glioma, MGMT methylation status and SIRI at 2-year, 3-year and 4-year in operable grade III glioma patients.

**Figure 5:**
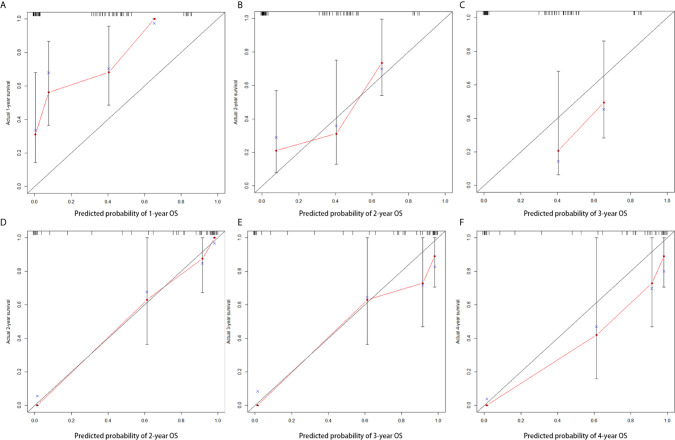
Calibration curve for predicting the 1-year, 2-year and 3-year OS **(A–C)** in patients with grade IV glioma and for predicting the 2-year, 3-year and 4-year OS **(D–F)** in patients with grade III glioma.

**Figure 6:**
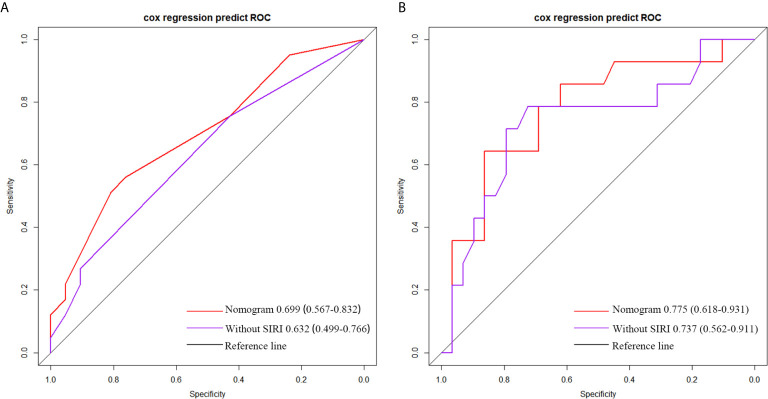
The roc analysis of the prediction prognosis ability of nomogram and predictive factors without SIRI in **(A)** grade IV and **(B)** grade III glioma patients.

Age, extent of resection, number of gliomas, MGMT methylation status and preoperative SIRI levels were important factors related to the prognosis of high grade gliomas, so they were included in the nomogram. In grade III gliomas, we also included the histology in the nomogram. **Figure 4A** shows the prognostic nomogram for survival rate of patients with grade IV glioma at 1, 2 and 3 years. The c-index of the established nomogram was 0.781 (95% CI: 0.705-0.857). The calibration curve showed that when predicting the 2-year survival rate, the prediction and observation showed good agreement (**Figures 5A–C**), indicating that the nomogram had reliable repeatability. ROC analysis further verified the predictive value of the nomogram. In the analysis of the OS of patients with grade IV glioma, the AUC of the nomogram was 0.699 (95%CI: 0.567-0.832), which was higher than the AUC of age, number of glioma and MGMT methylation status (AUC = 0.632, 95%CI: 0.499-0.766] (**Figure 6A**), indicating that the nomogram could predict the prognosis more accurately in operable grade IV glioma patients. **Figure 4B** shows the prognostic nomogram for survival rate of patients with grade III glioma at 2, 3 and 4 years. The c-index was 0.879 (95% CI: 0.779-0.979) and the calibration curve showed good consistency (**Figures 5D–F**). ROC analysis showed the AUC of the nomogram of grade III glioma was 0.775 (95%CI: 0.618-0.931), which was higher than the AUC of predictive factors without SIRI (AUC = 0.737, 95%CI: 0.562-0.911) (**Figure 6B**). This also indicated that the good predictive ability of the nomogram in grade III glioma patients.

## Discussion

In recent years, the role of systemic inflammatory markers (such as NLR, MLR, PLR, etc.) in predicting the prognosis of patients with malignant tumors has been discovered (7–12). Some studies found that NLR had predictive value in glioma patient (10, 18, 20). However, no consensus has been reached (21–23). The prognostic value of SIRI as a new indicator was first found in patients with pancreatic cancer (24). In recent years, more and more studies have confirmed the predictive role of systemic inflammatory response index (SIRI) in pancreatic cancer (24), cervical cancer (13), metastatic colorectal cancer (14), breast cancer (15), esophageal cancer (25), nasopharyngeal carcinoma (26), stomach cancer (27), gallbladder cancer (28) and other types of malignant tumors. In glioma, NLR was also found to have predictive value (10, 18, 20). However, the predictive effect of SIRI in patients with high-grade glioma is unclear. This study mainly explored the predictive value of preoperative SIRI, NLR, MLR, and PLR in high-grade gliomas. This study only included patients with high-grade glioma, because there were too many confounding factors for low-grade gliomas. In addition, due to the development of molecular pathology classification, some high-grade gliomas may be classified as low-grade in the past years. And this study only included patients who received standardized postoperative adjuvant radiotherapy and chemotherapy. The predictive value of preoperative inflammatory markers (SIRI, NLR, MLR and PLR) can be better reflected after excluding the influence of therapeutic factors.

In grade III and IV gliomas, the frequency of MGMT methylation (53% and 34%, 90respectively) and IDH mutations (88% and 32%, respectively) reported in our study was similar to the previously reported incidence of biomarkers in high-grade gliomas (29, 30). Our results confirmed the known influencing factors for OS of high-grade gliomas, such as age, histological type, number of gliomas, extent of resection, MGMT methylation status, etc. (30–35). For the IDH mutation status, we only observed trends related to OS benefits. The possible reason was that the number of IDH wild-type patients we included was small. The focus of the study was to explore the predictive value of SIRI in high-grade gliomas. Our results were consistent with other retrospective studies exploring the predictive value of SIRI in malignant tumors (13–15, 24–28). Our study found that preoperative SIRI was an independent prognostic factor for high-grade glioma. Our results showed that lower preoperative SIRI was an independent predictor of better OS in complete dataset, matched dataset, in the IDH mutant subgroup of grade III glioma, as well as in the IDH wild-type subgroup of grade IV glioma. However, in IDH mutant-type grade IV gliomas subgroup, lower SIRI only showed a tendency to obtain better OS. This may be because IDH mutant-type of grade IV gliomas may had a better prognosis, partially offsetting the predictive advantage of SIRI, and the number of IDH-mutated grade IV gliomas patients included was small (n=20). The above factors may affect the reliability of statistical results. We would further expand the sample size to explore the role of SIRI in such patients. We also included five IDH wild-type patients in grade III glioma. With the development of molecular pathology, it is necessary to further analyze the IDH wild-type grade III glioma patients and identify those who are classified as grade IV glioma patient. In future research and clinical work, we will include more patients and evaluate the prognostic value of SIRI in the subgroup. It was worth noting that some groups showed no significant difference in OS benefit for patients with lower preoperative SIRI in univariate analysis, but in multivariate analysis, patients with lower SIRI showed significant OS benefit. This may be because the OS was not only affected by SIRI, but also by other factors. When the influence of other factors is controlled by multivariate analysis, the significance of preoperative SIRI was revealed. Through ROC curve, we also found that SIRI was more accurate in predicting prognosis than PLR and MLR in high-grade glioma. This finding was consistent with the conclusions found in other malignant tumors (18, 22). And the predictive value of SIRI was even higher than NLR in grade IV gliomas. We would include more patients in the future to verify this finding. The nomogram based on SIRI showed good predictive performance, suggesting that SIRI, as a simple and cheap indicator, could predict the prognosis of high-grade glioma well. And the AUC of the nomogram based on SIRI was higher than that without SIRI. This suggested that the nomogram combined with preoperative SIRI was better than traditional biomarkers.

Prognostic judgment based on preoperative SIRI may be able to guide surgical decision making. Surgery is the main treatment for high-grade gliomas. In general, maximal resection of the tumor is conducive to a better prognosis for patients with high-grade glioma (36, 37). However, in some glioma patients with poor prognosis, the extent of surgical resection is not the main factor affecting the prognosis (38, 39). For glioma patients with different prognosis, surgical strategies may be different. It was found that in glioblastoma, the maximum contrast-enhanced (CE) tumor resection for elderly patients, and the maximum resection of CE tumor with additional maximum resection of the non-contrast-enhanced (NCE) tumor for young patients (≤65 years old) were related to better OS (40). SIRI, as a preoperative indicator that could predict the prognosis of patients with high-grade glioma, was expected to become one of the markers for determining the extent of surgical resection. In IDH wild-type grade IV glioma, patients with higher SIRI tended to have a poor prognosis. Surgeons may choose to limit the extent of resection, protect important neurological function and improve the patient’s quality of life. In IDH mutant-type grade III gliomas, patients with lower SIRI tend to have a longer survival, and for these patients, it may be possible to consider maximal extent of resection to prolong the survival period.

Our findings suggested that SIRI was promising in high-grade gliomas. With the development of molecular detection methods, the exact molecular pathological diagnosis of patients may change in the future. We will continue to follow up and further optimize the nomogram to predict patient prognosis more reliably.

These inflammatory markers may affect the prognosis of patients with malignant tumors in many ways. Neutrophils can promote the formation of the inflammatory microenvironment, inhibit lymphocyte activity, inhibit T cell response, and promote angiogenesis in various ways, thereby promoting tumor growth and metastasis, and exerting immunosuppressive effects (41–44). Lymphocytes play an important role in the body’s anti-tumor immunity. It exerts anti-tumor effects by inhibiting tumor proliferation and metastasis (45, 46). Monocytes (especially tumor-associated macrophages, TAMS) can promote tumor growth and metastasis, and can induce macrophages to promote angiogenesis through the expression of CXCL1 and CXCL8 (47, 48). Platelets can promote tumor growth, metastasis, and tumor angiogenesis, leading to tumor progression (47–50). Therefore, many inflammatory markers have been found to have certain predictive value in a variety of tumors. However, due to the existence of the blood-brain barrier and the suppressive immune microenvironment in high-grade gliomas (51), the prognostic value of some inflammatory markers in high-grade gliomas needed to be further explored.

However, this study had certain limitations (1). As a single-center, retrospective study, it had some inherent limitations. There may be selection bias, the number of patients was limited, and the follow-up time was not long enough. More researches were needed to verify our findings. (2) According to the latest progress in molecular pathology of glioma, the prognosis of patients with high-grade glioma was related to the molecular status of the tumor. We will continue to follow up and collect more patients with more complete tumor molecular and genetic test results to verify our findings. (3) Although the internally verified nomograms suggest that the predicted and observed OS had consistency, external verification was still needed to confirm whether our findings are generally applicable.

## Conclusions

The prognostic value of SIRI was reflected in high-grade gliomas. The nomogram covering SIRI predicted the survival rate of patients with high-grade glioma more reliably. This could help clinicians formulate a more reasonable individualized treatment plan.

## Data Availability Statement

The original contributions presented in the study are included in the article/**Supplementary Material**, further inquiries can be directed to the corresponding author.

## Ethics Statement

The studies involving human participants were reviewed and approved by the Ethics Committee of the First Affiliated Hospital of Chongqing Medical University. The ethics committee waived the requirement of written informed consent for participation. Written informed consent was obtained from the individual(s) for the publication of any potentially identifiable images or data included in this article.

## Author Contributions

(I) Conception and design: All authors; (II) Administrative support: None; (III) Provision of study materials or patients: Q He, QL Ren; (IV) Collection and assembly of data: Q He, LH Li; (V) Data analysis and interpretation: Q He, QL Ren; (VI) Manuscript writing: All authors. All authors contributed to the article and approved the submitted version.

## Conflict of Interest

The authors declare that the research was conducted in the absence of any commercial or financial relationships that could be construed as a potential conflict of interest.
